# Attitudes towards genetic testing and information: does parenthood shape the views?

**DOI:** 10.1007/s12687-020-00462-8

**Published:** 2020-04-04

**Authors:** Antti Saastamoinen, Virva Hyttinen, Mika Kortelainen, Juho Aaltio, Mari Auranen, Emil Ylikallio, Tuula Lönnqvist, Markus Sainio, Anu Suomalainen, Henna Tyynismaa, Pirjo Isohanni

**Affiliations:** 1 Finnish Competition and Consumer Authority, Helsinki, Finland; 2 VATT Institute for Economic Research, PO Box 1279, 00100 Helsinki, Finland; 3 Department of Health and Social Management, University of Eastern Finland, Kuopio, Finland; 4 Turku School of Economics, University of Turku, Turku, Finland; 5 Research Programs Unit, Stem Cells and Metabolism, University of Helsinki, Helsinki, Finland; 6 Clinical Neurosciences, Neurology, Helsinki University Hospital, University of Helsinki, Helsinki, Finland; 7 Department of Child Neurology, Children’s Hospital, Helsinki University Hospital, University of Helsinki, Helsinki, Finland; 8 Neuroscience Center, University of Helsinki, Helsinki, Finland; 9 Department of Medical and Clinical Genetics, University of Helsinki, Helsinki, Finland

**Keywords:** Genetic information, Parental attitudes, Survey, Finland

## Abstract

This study examines how parents of pediatric patients might differ in their views and attitudes towards genetic technology and information when compared to adult patients. There is surprisingly little evidence on how parents compare to other parts of population in their attitudes. Previous empirical studies often relate health-related preferences and attitudes to factors such as age, education, and income instead of parental status, thus evading comparison of parents to others as health-related decision makers. Findings related to the parental status can be useful when implementing genetic technology in clinical practice. We conducted a survey of views on genetic technology and information for groups of adult neurology patients (*n* = 68) and parents of pediatric neurology patients (*n* = 31) to shed some light on this issue. In addition to our own survey instrument, we conducted other surveys to gain insight on psychosocial factors that might affect these attitudes. The results suggest that parents are more concerned about their children’s genetic risk factors when compared to the attitudes of adult patients about their own risk. For both groups, negative emotional state was associated with more concerns towards genetic information. Our study provides insights on how parental views might affect the acceptance of genetic technology and information.

**Electronic supplementary material:**

The online version of this article (10.1007/s12687-020-00462-8) contains supplementary material, which is available to authorized users.

## Introduction

Genetic testing plays an important role for the diagnosis of genetic conditions (Stark et al. 2018). In addition to prognostic information, early diagnosis can have high diagnostic and clinical utility (Stark et al. 2018). The perceived utility of genetic testing extends often even beyond clinically actionable results as people perceive also informational value in test results (Halverson et al. 2016). Genetic information can influence e.g. families’ decision-making, such as parental reproductive planning (Malek et al. 2017; Stark et al. 2019). The benefits that the new personalized medicine paradigm promises to accrue are seen mainly come through the increased utilization of detailed health-related information, not only in treatment but also possibly in fostering health behavior change (see, e.g., McBride et al. 2010).

Last 20 years have thus witnessed a plethora of research on attitudes towards genetic information (see, e.g., Aro et al. 1997; Jallinoja et al. 1998; Jallinoja and Aro 2000; Morren et al. 2007; Singer et al. 2008; Vermeulen et al. 2013; Henneman et al. 2013; Oliveri et al. 2016; LePoire et al. 2018; Vornanen et al. 2018). Generally, attitudes in public opinion surveys have been found to be rather positive towards the utilization of genetic information in personalized medicine (e.g., LePoire et al. 2018). While general public opinion surveys often relate attitudes to individual characteristics such as gender, age and educational status, parental status has received rather scant attention. Parental aspect is anyhow one of the key components when considering the success of genetic counseling in pediatrics (Fernandez et al. 2014; Sapp et al. 2014).

In recent years, multiple studies have examined views on genetic information, technology, and the counseling process also from the viewpoint of parents (see, e.g., Tercyak et al. 2011; Madeo et al. 2014; Goldenberg et al. 2014; Fitzgerald-Butt et al. 2014; Krabbenborg et al. 2016a; Malek et al. 2017; Oberg et al. 2018). Overall, the studies point out that parents have interest and positive attitudes towards genetic technology and information (e.g., LePoire et al. 2018). Although relatively good number of studies now document the parental views on genetics, to our knowledge, much less is known on how parents fare against other groups when they consider their sentiments towards genetic medicine. On the other hand, studies concentrating on parental populations typically evade comparisons as they examine parents in isolation. Parents however may have differing needs in terms of information and counseling process as suggested by Krabbenborg et al. (2016a). In a parallel study, Krabbenborg et al. (2016b) focused on psychosocial reactions of parents when obtaining results of different degree of certainty and found that more conclusive results seemed to improve parents’ capabilities to adjust to the situation. Malek et al. (2017) again found that parents among pediatric cancer patients perceive that genomic information has psychological and pragmatic utility, even in the absence of clinical utility. In more general terms beyond genetic testing, for example, Madeo et al. (2012) have studied the factors that are associated with greater perceived uncertainty among parents of children with undiagnosed medical conditions. According to their results, parents experiencing less control over the illness of their child perceive higher uncertainty. Given all this, there are reasons to believe that there is a gap in the literature which would explicitly consider parental attitudes in contrast to attitudes of other groups, such as adult patients.

Besides the apparent empirical examination of comparing parental views to the views of adult patients, our study can also be placed into the context of wider literature related on how people react to (medical) information. First, we can relate our study to the concept of information avoidance which can be defined as a situation where an individual chooses not to obtain the information he or she knows exists (see, e.g., Golman et al. 2017). In the genetic testing context, for example, Clift et al. (2015) and Taber et al. (2015) have found results suggesting the prevalence of information avoidance behavior. In other contexts, information avoidance has been observed in cases such as overall cancer risk (Emanuel et al. 2015; Vrinten et al. 2018) and breast cancer screening take-up (Goldzahl 2017). The information acquisition or avoidance behavior has been formally studied using, for example, the decision models that incorporate anticipatory emotions (see, e.g., Caplin and Leahy 2001). In these models, uncertain health outcomes may cause anxiety for the decision maker and by either obtaining or avoiding information, the decision maker can try to avoid anxiety. For example, Barbour et al. (2012) have documented that maintaining hope or denial of bad health states can both act as motivators to avoid health information. Information avoidance is also related to psychosocial factors such as stress. Vrinten et al. (2018) found that persons with higher levels of psychosocial stress avoid cancer information more likely. While previous studies have more often examined how genetic testing influences psychosocial or psychological wellbeing (e.g., Cameron and Muller 2009; Oliveri et al. 2018), less is known about how psychosocial factors are associated with the views of genetic information. A study by Waisbren et al. (2016) is one of the few previous studies, which suggested that parental stress is associated with greater interest in genomic sequencing of newborns.

Second, it is commonly understood that health decisions involve a significant intertemporal aspect as often the costs and benefits of health choices accrue at different times (see, e.g., Attema 2012). This can be especially so in the case of parents as their decisions concerning their children are generally long-term decisions. Lastly, it is well established that there is significant heterogeneity in risk preferences due to individual characteristics. For example, Görlitz and Tamm (2015) have suggested that parenthood induces more risk aversive behavior. Chaulk et al. (2003) have argued that social expectations about the parental behavior might alter the way parents respond to risk. While we do not explicitly study any of these mechanisms directly, our study sheds some light about the possibility of these mechanisms in action in the context of genetic medicine.

In this study, our interest is to reveal some possible differences between the parents of child patients and adult patients in their views towards genetic issues. Moreover, we study whether there are different psychosocial factors at play behind these views. To achieve this, we survey their views towards genetic information and technology with our own questionnaire. In addition, we administer other validated questionnaires to examine the possible psychosocial factors behind their perceived views (see, e.g., Cameron and Muller 2009). For adults, we administer RAND-36 (abbreviated RAND hereafter) and Beck’s Depression Inventory (abbreviated BDI hereafter) questionnaires. RAND is a widely applied 36-item questionnaire on mental and physical well-being of the respondent. BDI on the other hand is a common tool for screening symptoms of depression. For parents of child patients, we administer a questionnaire more suitable to describe their parental situation, namely Parenting Stress Index (abbreviated PSI hereafter) by PAR Inc. (see Abidin 1995), which measures stressful aspects of parenthood, including subscales of e.g. depression symptoms and feelings of isolation.

## Methods

### Study design

The survey data and the corresponding information consent forms were gathered as a part of a project which aims to examine the cost-effectiveness of whole-exome sequencing and its effects on the care trajectories of patients. Our study design incorporates two groups, parents of child patients and adult patients, which are compared to each other. As our aim is not to do a longitudinal analysis of attitudes, cross-sectional data should be sufficient to describe the possible differences between the groups and associations between psychosocial factors and views on genetic issues.

### Participants and procedures

The participants were recruited at the pediatric neurology clinic and the adult neurology clinic of Helsinki University Hospital. Both patient groups were selected for the study by specialist physicians. Patients presented with neurological diseases of unknown etiology and suspicion of genetic cause, thus qualifying for the sequencing study. Children in the study manifested with infantile-onset severe neurological diseases or childhood-onset progressive neurological disorders and adult patients presented with severe neuromuscular disorders, ataxia, hereditary spastic paraparesis, or early Parkinson’s disease. The recruitment was mainly conducted during years 2016–2017 with continuous recruitment of incoming patients. Hence, the sampling procedure is a type of convenience sampling in which new participants were recruited as physicians came across suitable participants within their clinics. Surveys were distributed in paper form only to those who agreed to participate (adults: *n* = 100; parents: *n* = 47) of which 68 adult patients and 31 parents of 29 child patients completed the surveys. All surveys were conducted before any of the patients had their genetic sequencing done. All index patients who got molecular genetic diagnosis or their caregivers were discussed on the heredity of the disease by neurology specialist, and all those patients or families were offered genetic counseling by a clinical genetics specialist. Of 19 pediatric patients with genetic diagnosis, 12 families got genetic counseling, 6 are still waiting for it, and one thought it was unnecessary. Of 20 adult patients with genetic diagnosis, 16 got genetic counseling by a clinical geneticist, two elderly patients with no affected relatives got genetic counseling by their treating neurologist, one is still waiting for it, and one patient could not be contacted after genetic diagnosis was found. Lastly, this study follows the ethical standards set out by the coordinating ethical committee of The Hospital District of Helsinki and Uusimaa.

### Measures

There are five survey instruments administered in this study. The main instrument is our own questionnaire which includes questions related to attitudes towards genetic information and technology. For ease of subsequent discussion, we use the shorthand name “MAIN” to refer to our own survey. Two versions of the main survey were distributed, one for adult patients (referred as MAIN-A) and one for parents of child patients (referred as MAIN-C) (see questions in Table 1). The MAIN-C version had different wording so that the questions were formulated from the point of view of a parent, whereas the MAIN-A survey asked questions from the point of view of adult patients themselves. Furthermore, for ethical reasons, it was considered that two of the questions (concerning treatable and non-treatable secondary findings) included in the adult patient survey MAIN-A (consisting of 15 questions in total) would cause unnecessary stress for the parents and hence they were excluded from MAIN-C survey (consisting of 13 questions in total). Because of these excluded questions, the numbering of the questions is slightly different in child patient surveys. Thus, we denote different questions by their number and the letter “A” or “C” to refer either to adult or child survey. Except for the small change in wording, the remaining set of questions asks the same issues from both groups.

**Table 1:** Questions of the MAIN-A and MAIN-C surveys

Q1A/C: My attitude towards the future is positive (trusting) although there would be significant uncertainty associated with the things important for me going well. (1) My attitude is very trusting (2) My attitude is somewhat trusting (3) My attitude is neither positive or negative (4) My attitude is somewhat negative (5) My attitude is very negative	
Q2A/C: I believe that genetic information improves my quality of life regardless of the nature of information. (1) Yes (2) No (.) Cannot say	
Q3A/C: I believe that genetic information improves the quality of life of my close ones regardless of the nature of information. (1) Yes (2) No (.) Cannot say	
Q4A/C: Obtaining test results on inheritable risk factors related to my own (Q4C: my child’s) health would be important regardless of the content of the test results. (1) Yes (2) No (.) Cannot say	
Q5A/C: Knowledge that according to the test I have (Q5C: my child has) genetic risk factors would cause me concern. (3) Would cause very much concern (2) Would cause some concern (1) Would not cause any concern (.) Cannot say	
Q6A/C: Knowledge that according to the test I have (Q6C: my child has) genetic risk factors would influence my decisions related to my work and other issues concerning my personal finances. (3) Would have large influence (2) Would have some influence (1) Would not have any influence (.) Cannot say	
Q7A: Would you like to hear about such secondary findings concerning your health, which increase the probability of some other heritable illness? Let us assume in this case that this illness IS treatable if such illness later occurs. (1) I would like to know about this secondary finding (3) I would not like to know about this secondary finding (2) Secondary finding would make no difference for me	
Q7C: Do you think it would be reasonable that people could obtain information about disease risk factors related to inheritable conditions as a part of the general healthcare? (1) Yes, everybody should be able to obtain information if they wish so (2) Yes, but information should be given only to those who physician thinks the information would be useful (3) No, this information should not be given as a part of general healthcare (.) Cannot say	
Q8A: Would you like to hear about such secondary findings concerning your health, which increase the probability of some other heritable illness? Let us assume in this case that this illness IS NOT treatable if such illness later occurs. (1) I would like to know about this secondary finding (3) I would not like to know about this secondary finding (2) Secondary finding would make no difference for me	
Q8C: Do you think that information on genotype can be used for medical research if identity is concealed? (1) Yes (2) No (.) Cannot say	
Q9A: Do you think it would be reasonable that people could obtain information about disease risk factors related to inheritable conditions as a part of the general healthcare? (1) Yes, everybody should be able to obtain information if they wish so (2) Yes, but information should be given only to those who physician thinks the information would be useful (3) No, this information should not be given as a part of general healthcare (.) Cannot say	
Q9C: Do you think that information on genotype can be used for other purpose than medical research (e.g. research on kinship and historical and societal research) if identity is concealed? (1) Yes (2) No (.) Cannot say	
Q10A: Do you think that information on genotype can be used for medical research if identity is concealed? (1) Yes (2) No (.) Cannot say	
Q10C: Given my own financial situation, I would be willing to make a significant monetary investment on genetic testing to find out the nature of my/my children’s disease. (1) Yes (2) No (.) Cannot say	
Q11A: Do you think that information on genotype can be used for other purpose than medical research (e.g. research on kinship and historical and societal research) if identity is concealed? (1) Yes (2) No (.) Cannot say	
Q11C: If I had the following two options, I would like that: (1) Test results are provided fast (some weeks) but the results are less comprehensive (2) Test results are comprehensive but obtaining them takes more time	
Q12A: Given my own financial situation, I would be willing to make a significant monetary investment on genetic testing to find out the nature of my/my children’s disease. (1) Yes (2) No (.) Cannot say	
Q12C: If I had the following three options, I would like that: (1) I tell about the results to my close relatives myself (2) I tell about the results to my close relatives with the help of my doctor (3) The results would not be told to close relatives	
Q13A: If I had the following two options, I would like that: (1) Test results are provided fast (some weeks) but the results are less comprehensive (2) Test results are comprehensive but obtaining them takes more time	
Q13C: Should my close relatives be offered a chance to check their own genotype for risk factors if my own test results indicate the possibility of heritable disease? (1) Should be directly offered the chance (2) Should be offered the chance from their separate request (3) Should not be offered this chance at all	
Q14A: If I had the following three options, I would like that: (1) I tell about the results to my close relatives myself (2) I tell about the results to my close relatives with the help of my doctor (3) The results would not be told to close relatives	
Q15A: Should my close relatives be offered a chance to check their own genotype for risk factors if my own test results indicate the possibility of heritable disease? (1) Should be directly offered the chance (2) Should be offered the chance from their separate request (3) Should not be offered this chance at all	

In the MAIN-surveys we do not construct any overall composite score from the responses, but we analyze mean scores from each question separately. The translations of survey forms from Finnish to English along with the scoring of each question are given in Online Resources 1 & 2. Note that in the scoring of MAIN surveys, besides actual missing values, the so called “Cannot say” options (opt-out option) will be neglected from the calculation of question specific scores.

Besides our own survey instrument, we also administered three additional questionnaires to gather information about the psychosocial and physical aspects of respondents’ well-being and to examine whether there are any specific factors that correlate with attitudes in our MAIN-surveys. For adult patients, we administered RAND and BDI questionnaires. When using RAND we focus on various subscales, such as “physical functioning,” “emotional well-being,” and “general health,” that can be calculated based on the responses. In these RAND subscales, higher values correspond to more favorable states. In BDI, however, higher values reflect a more depressive state of the respondent. A brief account of these scoring procedures of RAND and BDI are given in the Online Resource 3.

For the parents of child patients, we administered the PSI questionnaire. As earlier mentioned RAND and BDI measure individuals’ own health and well-being, PSI measures stressful aspects related to parenthood. Since PSI is a commercial product, we cannot describe the exact procedures of its scoring or the content of the survey form. What we can however state here is that similar to RAND, various subscales can be formed from the PSI responses. These subscales represent for example depression symptoms, demandingness of the child, and feelings of isolation. In addition, PSI allows the formation of a composite score which represents the overall burden of parenthood (total parental domain score). In PSI, higher scores refer to a more negative situation in terms of that subscale. That is, for example in the case of depression subscale, higher values correspond to a more depressive/dissatisfied state of that parent. In the interpretation of these subscales we follow the 3rd edition of PSI manual (see Abidin 1995).

### Data analysis

We analyze the possible differences between genders and between the groups of parents of child patients and adult patients in their responses in the MAIN surveys by a standard unpaired mean comparison *t* test with the assumption of unequal variances in the groups. Thus, we compare the average answer scores between these two groups in each of the questions. We also test age-related effects on the differences in views between groups by ANOVA. Since we interpret all the measures to be ordinal at most, we examine these relationships with a correlation measure suitable for ordinal data, namely Kendall’s tau-b correlation coefficient. In addition, we report Cramer’s V and the *p* value from the Pearson *χ*^2^ test of independence between the measures when we examine the relationship of respondent’s general attitude towards uncertainty in the future (Q1A/Q1C) of either MAIN-survey with other questions in the corresponding MAIN surveys. The statistical significance was set at *p* value < 0.05. All analyses were done using the Stata software.

## Results

### Study sample composition

The composition of the study sample is given in Table 2. In total, 68 adult patients completed the MAIN-A survey and 31 parents of 29 child patients completed the MAIN-C survey. Adult patients are on average older than the parents of child patients (*p* < 0.001). The slight majority of adult patients are females. Female patients are also somewhat older on average. Among parents, mothers are overrepresented as only 3 out of 31 parent respondents are males. There are also twice as many sons than daughters among child patients. The mean age of child patients was 6.95 years (SD 5.7).

**Table 2:** Sample characteristics

	*N* (%)	Mean age	SD	Median	Min	Max			
Adult patients	68 (100)	49.60	14.76	49.5	18	81			
Male	30 (44.12)	46.97	12.73	47	19	69			
Female	38 (55.88)	51.68	16.05	55	18	81			
							Child gender		
							Male	Female	Missing
Parents of child patients	31 (100)	37.29	7.17	37	26	57			
Male	3 (9.68)	37.33	5.69	39	31	42	2	1	0
Female	28 (90.32)	37.29	7.40	37	26	57	19	8	1
Child patients	29 (100)	6.95^*^	5.7	5	0.17	16			

*Age measured in full years. For two child patients, the information on age was missing. Gender information on one child was missing

### Differences between adult patients and parents of child patients on the views of genetic information

Table 3 shows the mean answer scores in adult patient and parent groups for comparable questions, along with number of respondents in each question. The frequency tables of answers for each question in the MAIN-A and MAIN-C surveys are given in Online Resources 4 & 5. In addition, Online Resource 6 reports the share of answers by different groups for each question.

**Table 3:** Mean answers by adult patients and parents of child patients

Adults				Parents					
MAIN-A	Mean	*N*	Cannot say (*N*)	MAIN-C	Mean	*N*	Cannot say (*N*)	Difference	*p* value
Q1A Attitude towards uncertainty	2.045	66	–	Q1C Attitude towards uncertainty	1.806	31	–	0.239	0.1047
Q2A Own QoL	1.078	51	16	Q2C Own QoL	1.136	22	9	− 0.058	0.4953
Q3A The QoL of the close ones	1.102	49	18	Q3C The QoL of the close ones	1.118	17	14	− 0.016	0.8661
Q4A Importance of test results on inheritable risk factors	1.048	63	4	Q4C Importance of test results on inheritable risk factors	1.036	28	3	0.012	0.7914
Q5A Concern towards the knowledge of genetic risk factors	1.803	61	5	Q5C Concern towards the knowledge of genetic risk factors	2.111	27	3	− 0.308	0.0351
Q6A Influence of genetic risk factors on work life and financial decisions	1.642	53	14	Q6C Influence of genetic risk factors on work life and financial decisions	1.692	26	5	− 0.050	0.7247
Q7A Treatable secondary findings	1.000	67	–						
Q8A Non-treatable secondary findings	1.269	67	–						
Q9A Attitude towards genetic information as a part of healthcare	1.323	65	2	Q7C Attitude towards genetic information as a part of healthcare	1.448	29	1	− 0.125	0.3291
Q10A Genetic privacy and medical use of results	1.000	63	4	Q8C Genetic privacy and medical use of results	1.000	30	1	0.000	–
Q11A Genetic privacy and other than medical use of results	1.050	60	7	Q9C Genetic privacy and other than medical use of results	1.080	25	6	− 0.030	0.6325
Q12A Monetary investments	1.356	45	22	Q10C Monetary investments	1.250	16	14	0.106	0.4342
Q13A Availability of test results	1.925	67	–	Q11C Availability of test results	1.742	31	–	0.183	0.0395
Q14A Informing relatives about test results	1.303	66	–	Q12C Informing relatives about test results	1.267	30	–	0.036	0.7443
Q15A Possibility of heritable disease	1.433	67	–	Q13C Possibility of heritable disease	1.516	31	–	− 0.083	0.4510

Comparable questions from MAIN-A (adult patient survey) and MAIN-C (parent survey) next to each other. “Mean” refers to the mean score in the corresponding question. “Difference” refers to the difference between the mean scores. “*N*” is number of respondents. Note that the sum of respondents may not be 68 (adults) or 31 (parents) as there can be either “Cannot say” or truly missing values which are not included in the calculations of mean score

*QoL* quality of life

We observe that adult patients have slightly more negative attitude towards uncertainty in the future than parents (Q1A vs. Q1C). When comparing the share of answers, less parents (0%) than adult patients (4.4%) consider their future to be somewhat negative. Parents express more concern about the possible genetic risk factors of their child than adult patients do of their own risk factors (Q5C vs. Q5A. About 22.6% of parents compared to 5.9% adults think that knowledge of possible genetic risk factors would cause concern very much. We also observe that adult patients were somewhat more willing to wait for more comprehensive results whereas parents expressed slight tendency to be more impatient to obtain the results (Q13A vs. Q11C). About 25.8% of parents compared to 7.4% of adults think that test results would be better provided fast and less comprehensive than more comprehensive but obtaining test results takes more time.

While adult patients were on average older than the parents of child patients, we tested if some of the differences on the views between the groups are driven by possible age-related effects. However, our analysis did not show age-related effects, while interaction terms between age and group variables were not statistically significant (data not shown).

We also analyzed how the general attitude towards uncertainty in the future (Q1A/C) was associated with the other questions in the MAIN survey in both groups (Table 4). Among adult patients, the association between the general attitude towards uncertainty (Q1A) and importance of obtaining the risk factors related to health (Q4A) was positive and thus indicates that negativity is associated with smaller importance placed on genetic information. However, this finding is driven by only very few respondents answering “No” as the vast majority of adult patients saw genetic information important regardless of the results. Parents who have more negative general attitude towards uncertainty in the future see that genetic information would improve the quality of life of their closest ones (Q3C). However, only about half of the parent sample had some clear opinion on this and the other half (*n* = 14) responded the “Cannot say” option, reducing the sample size in this question significantly. More negativity towards uncertainty in the future in parents is associated with the desire to make significant adjustments to work life and economic decisions when they learn the genetic risk factors (Q6C).

**Table 4:** Associations between “general attitude towards uncertainty in the future” and other MAIN-survey questions

MAIN-A	Ktau-b	Sig.	Cramer V	Chi2-p	MAIN-C	Ktau-b	Sig.	Cramer V	Chi2-p
Q1A Attitude towards uncertainty	1	0.000	1	0.000	Q1C Attitude towards uncertainty	1	0.000	1	0.000
Q2A Own QoL	0.21	0.114	0.28	0.266	Q2C Own QoL	0.05	0.867	0.28	0.436
Q3A The QoL of the close ones	0.21	0.134	0.27	0.312	Q3C The QoL of the close ones	− 0.52	0.041	0.57	0.066
Q4A Importance of test results on inheritable risk factors	0.29	0.017	0.44	0.008	Q4C Importance of test results on inheritable risk factors	0.07	0.763	0.13	0.782
Q5A Concern towards the knowledge of genetic risk factors	0.23	0.060	0.25	0.286	Q5C Concern towards the knowledge of genetic risk factors	0.25	0.175	0.20	0.692
Q6A Influence of genetic risk factors on work life and financial decisions	0.10	0.452	0.26	0.303	Q6C Influence of genetic risk factors on work life and financial decisions	0.52	0.007	0.44	0.039
Q7A Treatable secondary findings	*NA*	*NA*	*NA*	*NA*					
Q8A Non-treatable secondary findings	0.21	0.075	0.24	0.279					
Q9A Attitude towards genetic information as a part of healthcare	0.16	0.183	0.20	0.513	Q7C Attitude towards genetic information as a part of healthcare	− 0.35	0.053	0.31	0.233
Q10A Genetic privacy and medical use of results	*NA*	*NA*	*NA*	*NA*	Q8C Genetic privacy and medical use of results	*NA*	*NA*	*NA*	*NA*
Q11A Genetic privacy and other than medical use of results	− 0.00	1.000	0.10	0.896	Q9C Genetic privacy and other than medical use of results	0.07	0.768	0.22	0.543
Q12A Monetary investments	0.15	0.296	0.29	0.292	Q10C Monetary investments	0.17	0.551	0.45	0.195
Q13A Availability of test results	− 0.07	0.570	0.15	0.669	Q11C Availability of test results	0.07	0.734	0.14	0.749
Q14A Informing relatives about test results	− 0.02	0.900	0.21	0.395	Q12C Informing relatives about test results	− 0.01	0.977	0.24	0.508
Q15A Possibility of heritable disease	0.07	0.536	0.18	0.528	Q13C Possibility of heritable disease	0.11	0.537	0.12	0.792

Comparable questions from MAIN-A (adult patient survey) and MAIN-C (parent survey) next to each other

*NA* not available (no variation)

### Associations between psychosocial factors and the general attitude towards uncertainty in the future and views towards genetic information

Since there are only two significant correlations between the MAIN-A and BDI, we do not tabulate but report them here within the text. The general attitude towards uncertainty in the future (Q1A) was significantly correlated with BDI (0.40, *p* < 0.001). We see that higher negativity is positively correlated with the degree of depression symptoms. This gives us some assurance that attitude towards uncertainty in the future has succeeded to extract reasonably well at least some aspect of respondent’s world view. In addition, there was a weak but significant correlation between BDI and concerns to genetic information (Q5A: 0.23, *p* = 0.035). Note that BDI score is highly correlated also with the emotional well-being component of RAND36, with a coefficient of − 0.49, *p* < 0.001.

As we see from Table 5, most significant correlations with RAND components are negative. For example, when the emotional well-being in RAND scores increases, the score in general attitude towards uncertainty decreases, implying more positive attitude towards the future. Overall, attitude towards uncertainty in the future correlates significantly with many of the RAND components, indicating that attitude towards uncertainty (O1A) gives somewhat consistent view of respondent’s sentiment also when compared to RAND.

**Table 5:** Significant correlations between RAND components and MAIN-A questions

MAIN-A Question	RAND component	Correlation coefficient	*p* value
Q1 Attitude towards uncertainty	Physical role limitations	− 0.26	0.013
Q1 Attitude towards uncertainty	Energy/fatigue	− 0.33	0.001
Q1 Attitude towards uncertainty	Emotional well-being	− 0.38	0.000
Q1 Attitude towards uncertainty	Social functioning	− 0.28	0.006
Q1 Attitude towards uncertainty	General health	− 0.33	0.001
Q5 Concern towards the knowledge of genetic risk factors	Emotional role limitations	− 0.33	0.006
Q5 Concern towards the knowledge of genetic risk factors	Emotional well-being	− 0.23	0.031
Q6 Influence of genetic risk factors on work life and financial decisions	Physical functioning	0.34	0.004
Q6 Influence of genetic risk factors on work life and financial decisions	Emotional well-being	− 0.36	0.002

RAND components are subscores of RAND-36 survey measure and MAIN-A refers to our own survey for adult patients

Physical functioning component is positively correlated with the influence of genetic risk factors on the decisions related to work and other issues concerning respondent’s personal finances (Q6A). Intuitively those who are physically in better condition can also better adjust their lives in terms of work life and household economy. On the other hand, the ones who are emotionally better off (“Emotional well-being”) may see little need for changes, thus the negative correlation with the influence of genetic risk factors on work life and financial decisions (Q6A). Concern towards the knowledge of genetic risk factors (Q5A) correlates significantly with emotional components of RAND, implying that lower emotional well-being is associated with higher concerns due to genetic information.

In Table 6, we list all the significant correlations between MAIN-C questions and any of the subscale scores in PSI. However, in some of the questions, the correlation can be driven by only few observations (see Online Resource 5). We see that for parents with more depression symptoms and feelings of isolation, the knowledge of the genetic risk factors of their child is associated with higher impact of this information on their work life and economic decisions (Q6C). Q6C is also correlated with the total parental domain score, implying that overall challenges in parenting seem to be associated with higher influence of genetic information on work life and financial decisions.

**Table 6:** Significant correlations between MAIN-C questions and PSI subscales

MAIN-C	PSI subscale	Correlation coefficient	*p* value
Q6 Influence of genetic risk factors on work life and financial decisions	Isolation	0.42	0.103
Q6 Influence of genetic risk factors on work life and financial decisions	Depression	0.39	0.018
Q6 Influence of genetic risk factors on work life and financial decisions	Total parental domain score	0.37	0.023
Q10 Monetary investments	Demanding	− 0.45	0.045
Q13 Possibility of heritable disease	Depression	0.31	0.047
PSI subscales are subscores of PSI survey measure and MAIN-C refers to our own survey for the parents of child patients.			

We interestingly found a negative relationship between willingness to invest financially on genetic examinations (Q10C) and how parents feel that their child has been demanding in terms of care, but many (*n* = 14) answered “Cannot say.” Parents who have felt elevated stress about the demands of the child are in fact more likely to invest. The occurrence of depression symptoms is associated with a desire to limit the availability of testing to close relatives (Q13C).

### Gender differences among adult patients

As suggested, gender differences may drive the responses. Thus, we examine whether there are significant differences in responses between genders among adult patients. In Table 7, we see that mean answer scores in the MAIN-A survey do not statistically significantly differ between male and female respondents. Thus, it seems that at least in statistical sense neither of the genders seems to drive the overall adult patient responses too much. As a consequence, we consider that we can use the adult sample as a whole when we compare them to the group of parents, which is mostly females.

**Table 7:** Mean answers in MAIN-A questions by genders

	All		Males		Females			
Question	Mean	*N*	Mean	*N*	Mean	*N*	Difference	*p* value
Q1 Attitude towards uncertainty	2.045	66	2.103	29	2.000	37	0.10	0.605
Q2 Own QoL	1.078	51	1.143	21	1.033	30	0.11	0.209
Q3 The QoL of the close ones	1.102	49	1.056	18	1.129	31	− 0.07	0.379
Q4 Importance of test results on inheritable risk factors	1.048	63	1.037	27	1.056	36	− 0.02	0.731
Q5 Concern towards the knowledge of genetic risk factors	1.803	61	1.704	27	1.882	34	− 0.18	0.192
Q6 Influence of genetic risk factors on work life and financial decisions	1.642	53	1.720	25	1.571	28	0.15	0.330
Q7 Treatable secondary findings	1.000	67	1.0	29	1.000	38	0.00	–
Q8 Non-treatable secondary findings	1.269	67	1.2	29	1.289	38	− 0.05	0.769
Q9 Attitude towards genetic information as a part of healthcare	1.323	65	1.2	28	1.405	37	− 0.19	0.166
Q10 Genetic privacy and medical use of results	1.000	63	1.0	27	1.000	36	0.00	–
Q11 Genetic privacy and other than medical use of results	1.050	60	1.037	27	1.061	33	− 0.02	0.676
Q12 Monetary investments	1.356	45	1.318	22	1.391	23	− 0.07	0.618
Q13 Availability of test results	1.925	67	1.966	29	1.895	38	0.07	0.251
Q14 Informing relatives about test results	1.303	66	1.241	29	1.351	37	− 0.11	0.336
Q15 Possibility of heritable disease	1.433	67	1.483	29	1.395	38	0.09	0.481

“Mean” refers to the mean score in the corresponding question. “Difference” refers to the difference between the mean scores. “*N*” is number of respondents. Note that the sum of respondents may not be 68 as there can be either “Cannot say” or truly missing values which are not included in the calculations of mean score

Females show slightly more concern due to findings of genetic risk factors (Q5A) than males as higher values are related to more expressed concern. We see that genetic information would have slightly more influence on work life and financial decisions (Q6A) for males than females. Female respondents are somewhat more cautious in terms of obtaining information on genetic risk factors directly as a part of healthcare (Q9A). Women had somewhat more reservations in terms of informing relatives about the results or at least they prefer to have more help from physicians (Q14A). Note that in the outset of the question we explicitly defined also children as close relatives. However, all adult respondents were unanimous in their responses with the questions about treatable secondary findings (Q7A) and both groups about genetic privacy and medical use of results (Q10A and Q8C).

## Discussion

This study is one of the few studies that compare the views of parents of child patients on genetic information to some other group, in this study to the adult patients. Previous studies have found that in general parents have positive attitudes towards genetic information (e.g., Fitzgerald-Butt et al. 2014; Dodson et al. 2015; LePoire et al. 2018). In summary, our results suggest some statistically significant differences between parents and adult patients in their views towards genetic information and technology. Adult patients showed more negative attitudes towards uncertainty in future life, as parents, who on average were younger, may account also for their own (better) physical functionality when judging their future. On the other hand, parents seem to express more concern when they learn about the genetic risk factors of their child than what adult patients do when learning about their own risk. However, also many of the adult patients have probably children and can have fear of possible hereditary. Our results are still in line with the study by Aktan-Collan et al. (2011) who found that parents felt that discussing about children’s risk of hereditary cancer is the most difficult communication aspect compared to, e.g., discussing to parents’ own risk. Our results also indicate that while both groups on a whole prefer more comprehensive testing results over faster but less comprehensive ones, more parents would anyhow rather have results available faster. In both groups, negative emotional state was associated with more concerns towards genetic information.

Neither group seemed to show major signs of information avoidance tendencies as both groups overall saw that genetic information will improve the quality of their own and their close relatives’ lives. However, almost a half of parents of child patients could not respond to this question, which is understandable as genomic information is unknown before sequencing. The relatively large degree of similarity of views towards genetic issues observed in our study can be explained by the same initial situation as both groups were faced with a new genetic sequencing technology personally. Our study indicates that mostly emotional factors, such as depressive state of mind, seemed to be associated with higher concern about the genetic information in both groups. However, due to the small samples we must use caution in making too strong conclusions as some correlations related to psychosocial factors might be driven by very few observations. In other contexts, the higher levels of psychosocial stress have been found to be associated with cancer-related fear and information avoidance (Vrinten et al. 2018).

Related to the intertemporal aspect of decision making, parents seemed more forward looking than adult patients, thus suggesting different time preferences and planning horizons. On the other hand, impatience of parents to receive results would suggest their desire to resolve uncertainty faster. That is, parents seem to express some preference towards faster resolution of uncertainty regardless of resolution being somewhat incomplete. One possible explanation is that parents prefer earlier resolution in order to adjust health investment, work life, and financial decisions more effectively. Indeed, based on our results it seemed that parents were quite keen to obtain genetic information. Such observations would suggest that parents want to avoid the discomfort caused by uncertainty due to lack of information. This is in line with the study by Görlitz and Tamm (2015), who suggested that parenthood induces more risk aversive behavior. In addition, Malek et al. (2017) reported that parents perceive genomic information in pediatric cancer context to have significant benefits for themselves but also their other family members. Naturally many other factors could affect here as age and current health status (of the patient) related effects could also explain the differences in the observed time and coverage preferences. For example, Ferecatu and Önçüler (2016) have suggested that impatience decreases with age.

Parents’ willingness to make economic and work life adjustments was associated with more negative attitudes towards uncertainty in the future and depression symptoms. Similar association was observed with adult patients. Also having a more demanding child increased the likelihood that parents invested on finding genetic risk factors as parents who have had it easier with the child may not feel as pressed to find out possible genetic factors behind child’s behavior. This in line with the finding of a previous study that those parents whose child has two or more health conditions are more likely to be interested in genomic information (Dodson et al. 2015). Also, a study by Waisbren et al. (2016) found that parental stress was associated with greater interest in genomic sequencing. However, in our study, it is difficult to draw any exact conclusions from parents’ willingness to make economic and work life adjustments as we have no information on what type of financial constraints the groups on average have. Both groups were observed to be unanimous about the medical use of the results.

When comparing the results between adult patients and the parents of child patients we need to keep in mind the gender distributions as gender differences may drive the responses. Our study showed that female respondents among adult patients were somewhat more cautious in terms of obtaining genetic risk factors directly as a part of healthcare. Aro et al. (1997) and more recently Vermeulen et al. (2013) have found gender differences in attitudes towards genetic issues. The study by Aro et al. (1997) suggested that men are more favorable towards genetic testing. However, the views differ according to age and education. Vermeulen et al. (2013) found that females are more favorable to assessing family history. Our study also showed that women had somewhat more reservations in terms of disclosing the results to relatives. In general, it is often observed that support and guidance is needed in terms of information disclosing, especially when considering whether or not one should let their children know about the results (see, e.g., Tercyak et al. 2007; Cameron and Muller 2009; Madeo et al. 2014). Lastly, although it is possible that the gender of a child is associated with certain type of parental behavior and family development (see, e.g., Lundberg 2005), usually such association is considered for paternal behavior while our group of parents mostly consisted mothers.

### The limitations of study

As always, our study is not devoid of limitations. Empirical studies which attempt to elicit preferences, attitudes, and behavior or model relationship between these in the health context often have some situational limitations which undermine the generalizability of the results. One common limitation is that attitudes or preferences are elicited using hypothetical scenarios which may not reflect well the actual decision-making situation (see, e.g., Goldenberg et al. 2014). This may be the case also with some of our survey questions. Because of some pre-conditions for participation, small and highly selected samples pose a challenge in many studies. This limitation certainly concerns our study and thus the results should be interpreted accordingly. Small sample size and “Cannot say” answers can decrease statistical power to find some differences between the groups. A post hoc power analysis is not a univocally accepted method to validate research findings (see, e.g., Hoenig and Heisey 2001), so we acknowledge that a priori power analysis to determine a sufficient sample size for example might have been valuable, but on the other hand, convenience sampling in our study poses some challenges to this also as a priori a high degree of uncertainty is related to the achieved sample size in this approach. Given that the participants were selected by professionals within their own clinics obviously limits the generalizability of our sample. In addition, the sample only considers individuals with sufficient skills in Finnish language since the surveys were administered in Finnish.

Additional criticism towards our data set is that we conducted a survey of attitudes for a sample of individuals who are about to have firsthand experience on genetic sequencing technology, either as a parent or patient. Thus, respondents are probably inclined to have more positive views to begin with. In addition, the recruited child and adult patients in this study had different diseases being partly more severe among child neurology patients (e.g., encephalopathy), which might affect the attitudes of parents of child patients. However, comparison within a medical specialty (neurology) in a clinical setting might provide more relevant information from the perspective of clinical practice compared to large-scale general public opinion surveys in which parts of the study population may have rather limited interest towards the topic. It is also noteworthy that studies tend to be context specific and insights from one domain such as financial domain may be hard to transmit to health domain (see, e.g., Galizzi 2014). Even if we stay within the health domain, we might observe variations, for example, in risk attitudes (van der Pol and Ruggeri 2008; Ruggeri and van der Pol 2012). For example, Cameron et al. (2009) show that the way how people view genetic testing and their results depends on the perceived riskiness of the disease under testing. Thus, making wide ranging policy recommendations based on a few disease/condition specific empirical studies would not be advisable.

We also cannot infer the overall level of understanding that the respondents possess on the issues of inheritance and genetics (see, e.g., Henderson and Maguire 2000). Significant differences in this understanding can shape attitudes accordingly. For example, knowledge of genetic testing can differ due to varying understanding of the role of genes in diseases (Henderson and Maguire 2000; Haga et al. 2013). Some individuals believe that genetic diseases are something that you cannot avoid if it is inherited, but some understand that you can also be carrier, not necessarily sufferer (Henderson and Maguire 2000). As said, in our setting, all respondents can be assumed to be relatively well informed about the nature of the sequencing that the patients are about to experience.

As our survey was built to indicate views on wide variety of issues related to the genetic topics, typical considerations of validity and reliability focusing on the ability of the instrument to measure some single underlying construct were not applied in this context. Thus, it was considered that slightly differing surveys for the two groups would not pose a challenge for our analysis. Anyhow, since the condition of their child is quite sensitive issue for the parents, we saw that administering a slightly modified survey would better elicit their views than distributing the exact same form than what adult patients received. Furthermore, given that the survey was built only for the specific purpose of this study, we considered that an internal evaluation of the appropriateness of the survey form among research group members was sufficient. We however acknowledge that in possible future analyses of the issue, the survey should be developed further. We cannot for example rule out that the wording of the questions might have some effect on the answers. Lastly, while we know how many of those who agreed to participate to the study ultimately filled the forms, we have no information on how many declined to participate.

### Implications for practice

According to our results, the counseling process related to new genetic technologies should account for the different views that parents of child patients and adult patients have towards genetic information. Thus, the counseling process might need to be adapted to cover wider support needs than just the immediate clinical support. The situations of parents may differ with respect to how demanding the child is, thus suggesting that the counseling might need to be adjusted accordingly.

### Possible avenues for future research

Many aspects of methodology and data in our study could be improved upon. For example, our comparison could include a non-treatment group who is not going to experience genetic sequencing at all. Our study also suggests some interesting themes for future research. There is some indication that the social isolation and feelings of parental failure/incompetence are possibly fruitful avenues to study further in this context. For example, Krabbenborg et al. (2016b) identified some parents felt certain type of isolation after receiving genetic testing results as the results acted as a sort of label on their child. Another topic that would warrant a further study relates to the willingness of parents to invest time, money, and effort to genetic examinations. This would require detailed background information on the financial situation of different households.

## Conclusions

In this paper, we have studied how the attitudes of parents of child patients towards genetic information may differ from adult patients. In general, our study provides insights on possible factors which could have an impact on parental decision making in the genetic medicine context. Our study hints that parents may differ as users of genetic information from the others. We also observed that negative emotional state was associated with more concerns towards genetic information. From the perspective of health policy aiming to foster acceptance of genetic technology, we suggest that based on our results, parental aspect of decision making should be recognized.

## Electronic supplementary material

ESM 1 (DOCX 56 kb)
