# Supplementary material for: Attitudes towards genetic testing and information: does parenthood shape the views?
Source: J Community Genet. 2020 Apr 4;11(4):461–73. doi: 10.1007/s12687-020-00462-8 (PMC7475141; doi:10.1007/s12687-020-00462-8)
Supplement: Supplementary file 1 — (DOCX 56 kb) [file 12687_2020_462_MOESM1_ESM.docx]

**Attitudes towards genetic testing and information: Does parenthood shape the views?**

**Journal of Community Genetics**

Authors: Antti Saastamoinen, Virva Hyttinen, Mika Kortelainen, Juho Aaltio, Mari Auranen, Emil Ylikallio, Tuula Lönnqvist, Markus Sainio, Anu Suomalainen, Henna Tyynismaa, Pirjo Isohanni

Corresponding author: Virva Hyttinen, VATT Institute for Economic Research, virva.hyttinen@uef.fi

________________________________________________________________________________

# Online Resource 1: Translation of the MAIN-A survey form

This is the translation of MAIN-A survey form made by the authors. In addition of the translation, we have added the scoring of answer options that is used in the analysis. To highlight the fact that these scores are not visible in the original form, the scores have been denoted with red color. In addition, we have denoted the scoring for “Cannot say” options with period as these options received no scores. The text here obviously is not part of the original form either. The actual form begins after the cutoff line.

RESEARCH PROJECT

NEUROGENOMICS FINLAND

*Neurogenomics in routine diagnostics of children and adults: the effects on treatment and cost-effectiveness.*

**SURVEY FORM FOR ADULT PATIENTS**

**General instructions**

With this survey form we aim to study your views and attitudes towards genetic information, testing of inherited risks, and the use of this information. Questions are at very general level and the aim is not to extract detailed information about your health state. The information gathered from this survey is dealt as securely as any other information in this project. There are **15** questions in this survey and we request you to answer each question by marking box next to your choice or writing the answer to the space reserved to it. Please read the questions carefully and choose the option that best describes your view.

**BACKGROUND INFORMATION**

Here we ask some background information from you.

**Gender**

Male □

Female □

**Age ______**

**QUESTIONS**

**QUESTION 1
General attitude towards uncertainty**

In this question we ask about your general attitudes towards uncertainty in future life. Uncertainty can be related to any are of life, such as family relations, your economic situations, of health condition. With the help of this question we seek to examine whether different attitudes towards uncertainty in general might explain your attitudes towards genetic information. We ask you to answer in a similar way what would have been the situation had you not been part of this study (i.e. you would not have the information on your possible health condition you currently have).

1. **My attitude towards the future is positive (trusting) although there is would be significant uncertainty associated with the things important for me going well.**
2. My attitude is very trusting □
3. My attitude is somewhat trusting □
4. My attitude is neither positive or negative □
5. My attitude is somewhat negative □
6. My attitude is very negative □

**QUESTIONS 2 and 3
The impact of genetic information on quality of life**

With these questions we aim to examine how you see that genetic information in general would affect to the quality of life of you or your close ones. With genetic information we mean both information related to inheritable diseases and other genetic information such as information on kinship. With these questions we aim to understand your general attitude towards genetic information.

1. **I believe that genetic information improves my quality of life regardless of the nature of information.**

**(1)** Yes □
**(2)** No □

**(.)** Cannot say □

1. **I believe that genetic information improves the quality of life of my close ones regardless of the nature of information.**

**(1)** Yes □
**(2)** No □

**(.)** Cannot say □

**QUESTIONS 4-6**

With these questions we aim to examine how important you consider genetic information on inheritable risk factors is.

1. **Obtaining test results on inheritable risk factors related to my own health would be important regardless of the content of the test results.**

**(1)** Yes □
**(2)** No □

**(.)** Cannot say □

1. **Knowledge that according to the test I have genetic risk factors would cause me concern.**

**(3)** Would cause very much concern □

**(2)** Would cause some concern □
**(1)** Would not cause any concern □

**(.)** Cannot say □

1. **Knowledge that according to the test I have genetic risk factors would influence my decisions related to my work and other issues concerning my personal finances.**

**(3)** Would have large influence □

**(2)** Would have some influence □

**(1)** Would not have any influence □

**(.)** Cannot say □

**Questions 7 & 8
Secondary findings**

With questions 7 and 8 we aim to examine your attitude towards secondary findings of genetic testing. With the term *secondary finding* we mean such genetic findings which relate to your overall health but not to the disease under examination.

1. **Would you like to hear about such secondary findings concerning your health which increase the probability of some other heritable illness? Let us assume in this case that this illness IS treatable if such illness later occurs.**

**(1)** I would like to know about this secondary finding □

**(3)** I would not like to know about this secondary finding □

**(2)** Secondary finding would make no difference for me □

1. **Would you like to hear about such secondary findings concerning your health which increase the probability of some other heritable illness? Let us assume in this case that this illness IS NOT treatable if such illness later occurs.**

**(1)** I would like to know about this secondary finding □

**(3)** I would not like to know about this secondary finding □

**(2)** Secondary finding would make no difference for me □

**QUESTIONS 9-12
General attitude towards genetic testing and the use of test results**

With these questions we aim to examine your general attitude towards genetic testing. Please note that in these questions we do not refer to your current state of health or any test results concerning you but the purpose is to understand your views in general.

1. **Do you think it would be reasonable that people could obtain information about disease risk factors related to inheritable conditions as a part of the general healthcare?**

**(1)** Yes, everybody should be able to obtain information if they wish so □

**(2)** Yes, but information should be given only to □

those who physician thinks the information would be useful

**(3)** No, this information should not be □

given as a part of general healthcare

**(.)** Cannot say □

1. **Do you think that information on genotype can be used for medical research if identity is concealed?**

**(1)** Yes □
**(2)** No □

**(.)** Cannot say □

1. **Do you think that information on genotype can be used for other purpose than medical research (e.g. research on kinship and historical and societal research) if identity is concealed?**

**(1)** Yes □
**(2)** No □

**(.)** Cannot say □

1. **Given my own financial situation, I would be willing to make a significant monetary investment on genetic testing to find out the nature of my/my children’s disease.**

**(1)** Yes □
**(2)** No □

**(.)** Cannot say □

**QUESTIONS 13-15
Test results and handling of results**

With the next questions we aim to examine your views on how fast reporting and coverage you wish from test results, how willing to tell about the results to close relatives and whether you think the close relatives should be offered chance for testing based on your results. With close relatives we refer here to your parents, children, or siblings. Once again, with results we do not refer to your own possible results obtained within this research project, but we aim to understand your general views.

1. **If I had the following two options, I would like that:**

**(1)** Test results are provided
 fast (some weeks) □
 but the results are less comprehensive

**(2)** Test results are comprehensive
 but obtaining them □

takes more time

1. **If I had the following three options, I would like that:**

**(1)** I tell about the results to my close relatives myself □

**(2)** I tell about the results to my close relatives □

with the help of my doctor

**(3)** The results would not be □

told to close relatives

1. **Should my close relatives be offered a chance to check their own genotype for risk factors if my own test results indicate the possibility of heritable disease?**

**(1)** Should be directly offered the chance □

**(2)** Should be offered the chance □
 from their separate request

**(3)** Should not be offered this chance at all □

**THANK YOU FOR YOUR RESPONSES!**

# Online Resource 2: Translation of the MAIN-C survey form

This is the translation of MAIN-C survey form made by the authors. In addition of the translation, we have added the scoring of answer options that is used in the analysis. To highlight the fact that these scores are not visible in the original form, the scores have been denoted with red color. In addition, we have denoted the scoring for “Cannot say” options with period as these options received no scores. The text here obviously is not part of the original form either. The actual form begins after the cutoff line.

RESEARCH PROJECT

NEUROGENOMICS FINLAND

*Neurogenomics in routine diagnostics of children and adults: the effects on treatment and cost-effectiveness.*

**SURVEY FORM FOR PARENT OF PEDIATRIC PATIENTS**

**General instructions**

With this survey form we aim to study your views and attitudes towards genetic information, testing of inherited risks, and the use of this information. Questions are at very general level and the aim is not to extract detailed information about your child’s or own health state. The information gathered from this survey is dealt as securely as any other information in this project. There are **13** questions in this survey and we request you to answer each question by marking box next to your choice or writing the answer to the space reserved to it. Please read the questions carefully and choose the option that best describes your view.

**BACKGROUND INFORMATION**

Here we ask some background information concerning you and your child involved in this study.

**Parent information**

**Gender**

Male □

Female □

**Age ______**

**Child information**

**The gender of your child who is part of this study**

Male □

Female □

**The age of your child who is part of this study ______**

**QUESTIONS**

**QUESTION 1
General attitude towards uncertainty**

In this question we ask about your general attitudes towards uncertainty in future life. Uncertainty can be related to any are of life, such as family relations, your economic situations, of health condition. With the help of this question we seek to examine whether different attitudes towards uncertainty in general might explain your attitudes towards genetic information. We ask you to answer in a similar way what would have been the situation had your child not been part of this study (i.e. you would not have the information the possible health condition of your child you currently have).

1. **My attitude towards the future is positive (trusting) although there is would be significant uncertainty associated with the things important for me going well.**
2. My attitude is very trusting □
3. My attitude is somewhat trusting □
4. My attitude is neither positive or negative □
5. My attitude is somewhat negative □
6. My attitude is very negative □

**QUESTIONS 2 and 3
The impact of genetic information on quality of life**

With these questions we aim to examine how you see that genetic information in general would affect to the quality of life of you or your close ones. With genetic information we mean both information related to inheritable diseases and other genetic information such as information on kinship. With these questions we aim to understand your general attitude towards genetic information.

1. **I believe that genetic information improves my quality of life regardless of the nature of information.**

**(1)** Yes □
**(2)** No □

**(.)** Cannot say □

1. **I believe that genetic information improves the quality of life of my close ones regardless of the nature of information.**

**(1)** Yes □
**(2)** No □

**(.)** Cannot say □

**QUESTIONS 4-6**

With these questions we aim to examine how important you consider genetic information on inheritable risk factors of your child is.

1. **Obtaining test results on inheritable risk factors related to my child’s health would be important regardless of the content of the test results.**

**(1)** Yes □
**(2)** No □

**(.)** Cannot say □

1. **Knowledge that according to the test my child has genetic risk factors would cause me concern.**

**(3)** Would cause very much concern □

**(2)** Would cause some concern □
**(1)** Would not cause any concern □

**(.)** Cannot say □

1. **Knowledge that according to the test my child has genetic risk factors would influence my decisions related to my work and other issues concerning my personal finances.**

**(3)** Would have large influence □

**(2)** Would have some influence □

**(1)** Would not have any influence □

**(.)** Cannot say □

**QUESTIONS 7-10
General attitude towards genetic testing and the use of test results**

With these questions we aim to examine your general attitude towards genetic testing. Please note that in these questions we do not refer to the current state of health your child or any test results concerning your child but the purpose is to understand your views in general.

1. **Do you think it would be reasonable that people could obtain information about disease risk factors related to inheritable conditions as a part of the general healthcare?**

**(1)** Yes, everybody should be able to obtain information if they wish so □

**(2)** Yes, but information should be given only to □

those who physician thinks the information would be useful

**(3)** No, this information should not be □

given as a part of general healthcare

**(.)** Cannot say □

1. **Do you think that information on genotype can be used for medical research if identity is concealed?**

**(1)** Yes □
**(2)** No □

**(.)** Cannot say □

1. **Do you think that information on genotype can be used for other purpose than medical research (e.g. research on kinship and historical and societal research) if identity is concealed?**

**(1)** Yes □
**(2)** No □

**(.)** Cannot say □

1. **Given my own financial situation, I would be willing to make a significant monetary investment on genetic testing to find out the nature of my/my children’s disease.**

**(1)** Yes □
**(2)** No □

**(.)** Cannot say □

**QUESTIONS 11-13
Test results and handling of results**

With the next questions we aim to examine your views on how fast reporting and coverage you wish from test results, how willing to tell about the results to close relatives and whether you think the close relatives should be offered chance for testing based on your results. With close relatives we refer here to your parents, children, or siblings. Once again, with results we do not refer to the possible results of your child obtained within this research project, but we aim to understand your general views.

1. **If I had the following two options, I would like that:**

**(1)** Test results are provided
 fast (some weeks) □
 but the results are less comprehensive

**(2)** Test results are comprehensive
 but obtaining them □

takes more time

1. **If I had the following three options, I would like that:**

**(1)** I tell about the results to my close relatives myself □

**(2)** I tell about the results to my close relatives □

with the help of my doctor

**(3)** The results would not be □

told to close relatives

1. **Should my close relatives be offered a chance to check their own genotype for risk factors if my own test results indicate the possibility of heritable disease?**

**(1)** Should be directly offered the chance □

**(2)** Should be offered the chance □
 from their separate request

**(3)** Should not be offered this chance at all □

**THANK YOU FOR YOUR RESPONSES!**

# Online Resource 3: Scoring details of RAND-36, BDI, and PSI

Here we outline some details of how we have scored the auxiliary survey instruments that were administered along with our own survey.

**Parenting Stress Index (PSI), 3^rd^ edition**

Parenting Stress Index and the associated materials are commercial products which are under a license from PAR Inc ®. Thus we cannot describe here the content of the survey instrument or any of the materials in a very detailed manner. In scoring the survey, we have followed the instructions given in Abidin (1995; Parenting Stress Index 3^rd^ Edition, Professional Manual).

Additional information on PSI 3^rd^ edition is available from the following link:

<https://www.parinc.com/Products/Pkey/332> (last accessed 8.5.2018)

**Beck’s Depression Inventory (BDI)**

We have used a Finnish version of the Beck’s Depression Inventory. Questions concern different aspects of mental health (and related physical implications) such as sadness, feelings of failure, disappointment, and guilt, suicidal tendencies, appetite and weight loss. The questionnaire has 21 questions, with four answer options. Answer option are ordered in the sense that the first option corresponds to a position in which the respondent sees no change in his/her situation regarding the issue in question or lack of such state. The last answer option describes the most severe state in terms of the issue in question. The two answer options between these two more or less describe some intermediate states. The answers are scored such that the mildest answer option (first one) receives a score of zero, where the rest are scored with scores of 1,2, and 3. The total BDI-score is obtained as a sum of all scores. In our analysis we use the total BDI-score, although we have also categorized total scores into severity groups. In BDI, higher score indicates higher severity of depression symptoms.

**RAND-36 Questionnaire**

The version of RAND-36 that we use is the version 1.0. The scoring of RAND36 is done according to guidelines found from:

<https://www.rand.org/health/surveys_tools/mos/36-item-short-form/scoring.html> (last accessed 8.5.2018)

# Online Resource 4: MAIN-A answer frequencies

Note: “Missing” refers to truly missing values, “CNTS” refers to the “Cannot say” options.

|  |  |  |  |
| --- | --- | --- | --- |
| Q1A | Freq. | Percent | Cumul. |
| 1 | 16 | 23.53 | 23.53 |
| 2 | 34 | 50 | 73.53 |
| 3 | 13 | 19.12 | 92.65 |
| 4 | 3 | 4.410 | 97.06 |
| Missing | 2 | 2.940 | 100 |
| Total | 68 | 100 |  |
|  |  |  |  |
| Q2A | Freq. | Percent | Cumul. |
| 1 | 47 | 69.12 | 69.12 |
| 2 | 4 | 5.880 | 75 |
| CNTS | 16 | 23.53 | 98.53 |
| Missing | 1 | 1.470 | 100 |
| Total | 68 | 100 |  |
|  |  |  |  |
| Q3A | Freq. | Percent | Cumul. |
| 1 | 44 | 64.71 | 64.71 |
| 2 | 5 | 7.350 | 72.06 |
| CNTS | 18 | 26.47 | 98.53 |
| Missing | 1 | 1.470 | 100 |
| Total | 68 | 100 |  |
|  |  |  |  |
| Q4A | Freq. | Percent | Cumul. |
| 1 | 60 | 88.24 | 88.24 |
| 2 | 3 | 4.410 | 92.65 |
| CNTS | 4 | 5.880 | 98.53 |
| Missing | 1 | 1.470 | 100 |
| Total | 68 | 100 |  |
|  |  |  |  |
| Q5A | Freq. | Percent | Cumul. |
| 1 | 16 | 23.53 | 23.53 |
| 2 | 41 | 60.29 | 83.82 |
| 3 | 4 | 5.880 | 89.71 |
| CNTS | 5 | 7.350 | 97.06 |
| Missing | 2 | 2.940 | 100 |
| Total | 68 | 100 |  |
|  |  |  |  |
| Q6A | Freq. | Percent | Cumul. |
| 1 | 21 | 30.88 | 30.88 |
| 2 | 30 | 44.12 | 75 |
| 3 | 2 | 2.940 | 77.94 |
| CNTS | 14 | 20.59 | 98.53 |
| Missing | 1 | 1.470 | 100 |
| Total | 68 | 100 |  |
|  |  |  |  |
| Q7A | Freq. | Percent | Cumul. |
| 1 | 67 | 98.53 | 98.53 |
| Missing | 1 | 1.470 | 100 |
| Total | 68 | 100 |  |
|  |  |  |  |
| Q8A | Freq. | Percent | Cumul. |
| 1 | 57 | 83.82 | 83.82 |
| 2 | 2 | 2.940 | 86.76 |
| 3 | 8 | 11.76 | 98.53 |
| Missing | 1 | 1.470 | 100 |
| Total | 68 | 100 |  |
|  |  |  |  |
| Q9A | Freq. | Percent | Cumul. |
| 1 | 47 | 69.12 | 69.12 |
| 2 | 15 | 22.06 | 91.18 |
| 3 | 3 | 4.410 | 95.59 |
| CNTS | 2 | 2.940 | 98.53 |
| Missing | 1 | 1.470 | 100 |
| Total | 68 | 100 |  |
|  |  |  |  |
| Q10A | Freq. | Percent | Cumul. |
| 1 | 63 | 92.65 | 92.65 |
| CNTS | 4 | 5.880 | 98.53 |
| Missing | 1 | 1.470 | 100 |
| Total | 68 | 100 |  |
|  |  |  |  |
| Q11A | Freq. | Percent | Cumul. |
| 1 | 57 | 83.82 | 83.82 |
| 2 | 3 | 4.410 | 88.24 |
| CNTS | 7 | 10.29 | 98.53 |
| Missing | 1 | 1.470 | 100 |
| Total | 68 | 100 |  |
|  |  |  |  |
| Q12A | Freq. | Percent | Cumul. |
| 1 | 29 | 42.65 | 42.65 |
| 2 | 16 | 23.53 | 66.18 |
| CNTS | 22 | 32.35 | 98.53 |
| Missing | 1 | 1.470 | 100 |
| Total | 68 | 100 |  |
|  |  |  |  |
| Q13A | Freq. | Percent | Cumul. |
| 1 | 5 | 7.350 | 7.350 |
| 2 | 62 | 91.18 | 98.53 |
| Missing | 1 | 1.470 | 100 |
| Total | 68 | 100 |  |
|  |  |  |  |
| Q14A | Freq. | Percent | Cumul. |
| 1 | 46 | 67.65 | 67.65 |
| 2 | 20 | 29.41 | 97.06 |
| Missing | 2 | 2.940 | 100 |
| Total | 68 | 100 |  |
|  |  |  |  |
| Q15A | Freq. | Percent | Cumul. |
| 1 | 38 | 55.88 | 55.88 |
| 2 | 29 | 42.65 | 98.53 |
| Missing | 1 | 1.470 | 100 |
| Total | 68 | 100 |  |

# Online Resource 5: MAIN-C answer frequencies

Note: “Missing” refers to truly missing values, “CNTS” refers to the “Cannot say” options.

| Q1C | Freq. | Percent | Cumul. |
| --- | --- | --- | --- |
| 1 | 9 | 29.03 | 29.03 |
| 2 | 19 | 61.29 | 90.32 |
| 3 | 3 | 9.680 | 100 |
| Total | 31 | 100 |  |
|  |  |  |  |
| Q2C | Freq. | Percent | Cumul. |
| 1 | 19 | 61.29 | 61.29 |
| 2 | 3 | 9.680 | 70.97 |
| CNTS | 9 | 29.03 | 100 |
| Total | 31 | 100 |  |
|  |  |  |  |
| Q3C | Freq. | Percent | Cumul. |
| 1 | 15 | 48.39 | 48.39 |
| 2 | 2 | 6.450 | 54.84 |
| CNTS | 14 | 45.16 | 100 |
| Total | 31 | 100 |  |
|  |  |  |  |
| Q4C | Freq. | Percent | Cumul. |
| 1 | 27 | 87.10 | 87.10 |
| 2 | 1 | 3.230 | 90.32 |
| CNTS | 3 | 9.680 | 100 |
| Total | 31 | 100 |  |
|  |  |  |  |
| Q5C | Freq. | Percent | Cumul. |
| 1 | 4 | 12.90 | 12.90 |
| 2 | 16 | 51.61 | 64.52 |
| 3 | 7 | 22.58 | 87.10 |
| CNTS | 3 | 9.680 | 96.77 |
| Missing | 1 | 3.230 | 100 |
| Total | 31 | 100 |  |
|  |  |  |  |
| Q6C | Freq. | Percent | Cumul. |
| 1 | 10 | 32.26 | 32.26 |
| 2 | 14 | 45.16 | 77.42 |
| 3 | 2 | 6.450 | 83.87 |
| CNTS | 5 | 16.13 | 100 |
| Total | 31 | 100 |  |
|  |  |  |  |
| Q7C | Freq. | Percent | Cumul. |
| 1 | 17 | 54.84 | 54.84 |
| 2 | 11 | 35.48 | 90.32 |
| 3 | 1 | 3.230 | 93.55 |
| CNTS | 1 | 3.230 | 96.77 |
| Missing | 1 | 3.230 | 100 |
| Total | 31 | 100 |  |
|  |  |  |  |
| Q8C | Freq. | Percent | Cumul. |
| 1 | 30 | 96.77 | 96.77 |
| CNTS | 1 | 3.230 | 100 |
| Total | 31 | 100 |  |
|  |  |  |  |
| Q9C | Freq. | Percent | Cumul. |
| 1 | 23 | 74.19 | 74.19 |
| 2 | 2 | 6.450 | 80.65 |
| CNTS | 6 | 19.35 | 100 |
| Total | 31 | 100 |  |
|  |  |  |  |
| Q10C | Freq. | Percent | Cumul. |
| 1 | 12 | 38.71 | 38.71 |
| 2 | 4 | 12.90 | 51.61 |
| CNTS | 14 | 45.16 | 96.77 |
| Missing | 1 | 3.230 | 100 |
| Total | 31 | 100 |  |
|  |  |  |  |
| Q11C | Freq. | Percent | Cumul. |
| 1 | 8 | 25.81 | 25.81 |
| 2 | 23 | 74.19 | 100 |
| Total | 31 | 100 |  |
|  |  |  |  |
| Q12C | Freq. | Percent | Cumul. |
| 1 | 23 | 74.19 | 74.19 |
| 2 | 6 | 19.35 | 93.55 |
| 3 | 1 | 3.230 | 96.77 |
| Missing | 1 | 3.230 | 100 |
| Total | 31 | 100 |  |
|  |  |  |  |
| Q13C | Freq. | Percent | Cumul. |
| 1 | 15 | 48.39 | 48.39 |
| 2 | 16 | 51.61 | 100 |
| Total | 31 | 100 |  |

# Online Resource 6: Percentage shares of answer frequencies by adults and parents of child patients

| **Adults** | **%** | **Parents** | **%** |
| --- | --- | --- | --- |
| **Question 1A** |  | **Question 1C** |  |
| 1 | 23.5 | 1 | 29.0 |
| 2 | 50 | 2 | 61.3 |
| 3 | 19.1 | 3 | 9.7 |
| 4 | 4.4 | 4 | 0 |
| 5 | 0 | 5 | 0 |
| Missing | 2.9 | Missing | 0 |
| **Question 2A** |  | **Question 2C** |  |
| 1 | 69.1 | 1 | 61.3 |
| 2 | 5.9 | 2 | 9.7 |
| Cannot say | 23.5 | Cannot say | 29.0 |
| Missing | 1.5 | Missing | 0 |
| **Question 3A** |  | **Question 3C** |  |
| 1 | 64.7 | 1 | 48.4 |
| 2 | 7.4 | 2 | 6.4 |
| Cannot say | 26.5 | Cannot say | 45.2 |
| Missing | 1.5 | Missing | 0 |
| **Question 4A** |  | **Question 4C** |  |
| 1 | 88.2 | 1 | 87.1 |
| 2 | 4.4 | 2 | 3.2 |
| Cannot say | 5.9 | Cannot say | 9.7 |
| Missing | 1.5 | Missing | 0 |
| **Question 5A** |  | **Question 5C** |  |
| 1 | 23.5 | 1 | 12.9 |
| 2 | 60.3 | 2 | 51.6 |
| 3 | 5.9 | 3 | 22.6 |
| Cannot say | 7.4 | Cannot say | 9.7 |
| Missing | 2.9 | Missing | 3.2 |
| **Question 6A** |  | **Question 6C** |  |
| 1 | 30.9 | 1 | 32.3 |
| 2 | 44.1 | 2 | 45.2 |
| 3 | 2.9 | 3 | 6.4 |
| Cannot say | 20.6 | Cannot say | 16.1 |
| Missing | 1.5 | Missing | 0 |
| **Question 9A** |  | **Question 7C** |  |
| 1 | 69.1 | 1 | 54.8 |
| 2 | 22.1 | 2 | 35.5 |
| 3 | 4.4 | 3 | 3.2 |
| Cannot say | 2.9 | Cannot say | 3.2 |
| Missing | 1.5 | Missing | 3.2 |
| **Question 10A** |  | **Question 8C** |  |
| 1 | 92.7 | 1 | 96.8 |
| 2 | 0 | 2 | 0 |
| Cannot say | 5.9 | Cannot say | 3.2 |
| Missing | 1.5 | Missing | 0 |
| **Question 11A** |  | **Question 9C** |  |
| 1 | 83.8 | 1 | 74.2 |
| 2 | 4.4 | 2 | 6.4 |
| Cannot say | 10.3 | Cannot say | 19.4 |
| Missing | 1.5 | Missing | 0 |
| **Question 12A** |  | **Question 10C** |  |
| 1 | 42.7 | 1 | 38.7 |
| 2 | 23.5 | 2 | 12.9 |
| Cannot say | 32.4 | Cannot say | 45.7 |
| Missing | 1.5 | Missing | 3.2 |
| **Question 13A** |  | **Question 11C** |  |
| 1 | 7.4 | 1 | 25.8 |
| 2 | 91.2 | 2 | 74.2 |
| Missing | 1.4 | Missing | 0 |
| **Question 14A** |  | **Question 12C** |  |
| 1 | 67.7 | 1 | 74.2 |
| 2 | 29.4 | 2 | 19.4 |
| 3 | 0 | 3 | 3.2 |
| Missing | 2.9 | Missing | 3.2 |
| **Question 15A** |  | **Question 13C** |  |
| 1 | 55.9 | 1 | 48.4 |
| 2 | 42.6 | 2 | 51.6 |
| Missing | 1.5 | Missing | 0 |
